# Accelerated fracture healing in mice lacking the 5-lipoxygenase gene

**DOI:** 10.3109/17453674.2010.533931

**Published:** 2010-11-26

**Authors:** Michaele B Manigrasso, J Patrick O'Connor

**Affiliations:** Department of Biochemistry and Molecular Biology, UMDNJ-New Jersey Medical School and Graduate School of Biological Sciences, Newark, NJ, USA

## Abstract

**Background and purpose:**

Cyclooxygenase-2 (COX-2) promotes inflammation by synthesizing pro-inflammatory prostaglandins from arachidonic acid. Inflammation is an early response to bone fracture, and ablation of COX-2 activity impairs fracture healing. Arachidonic acid is also converted into leukotrienes by 5-lipoxygenase (5-LO). We hypothesized that 5-LO is a negative regulator of fracture healing and that in the absence of COX-2, excess leukotrienes synthesized by 5-LO will impair fracture healing.

**Methods:**

Fracture healing was assessed in mice with a targeted 5-LO mutation (5-LO^KO^ mice) and control mice by radiographic and histological observations, and measured by histomorphometry and torsional mechanical testing. To assess effects on arachidonic acid metabolism, prostaglandin E2, F2**α**, and leukotriene B4 levels were measured in the fracture calluses of control, 5-LO^KO^ COX-1^KO^, and COX-2^KO^ mice by enzyme linked immunoassays.

**Results:**

Femur fractures in 5-LO^KO^ mice rapidly developed a cartilaginous callus that was replaced with bone to heal fractures faster than in control mice. Femurs from 5-LO^KO^ mice had substantially better mechanical properties after 1 month of healing than did control mice. Callus leukotriene levels were 4-fold higher in mice homozygous for a targeted mutation in the COX-2 gene (COX-2^KO^), which indicated that arachidonic acid was shunted into the 5-LO pathway in the absence of COX-2.

**Interpretation:**

These experiments show that 5-LO negatively regulates fracture healing and that shunting of arachidonic acid into the 5-LO pathway may account, at least in part, for the impaired fracture healing response observed in COX-2^KO^ mice.

The molecular events that initiate and maintain bone fracture healing are poorly understood. Inflammation is an early physiological response to fracture and it has been postulated that signaling molecules produced during inflammation initiate the tissue regeneration pathway (Mountziaris and Mikos 2008, Cottrell et al. 2009).

Cyclooxygenase-2 (COX-2) is a critical, enzymatic regulator of inflammation (Simmons et al. 2004). Loss of COX-2 function dramatically impairs fracture healing in mice and rats (Simon et al. 2002). Fracture healing in COX-2 knock-out mice is characterized by formation of a small cartilaginous fracture callus with delayed, reduced, or no endochondral bone formation (Zhang et al. 2002). Pharmacological inhibition of COX-2 also severely impairs fracture healing (Simon and O'Connor 2007). Human studies examining the effects of non-steroidal anti-inflammatory drugs on fracture healing support the conclusions from animal studies (Burd et al. 2003).

Inflammatory stimuli activate phospholipase A_2_ to release arachidonic acid from membrane stores (Capper and Marshall 2001). In turn, arachidonic acid is converted into prostaglandins by COX-2 or into leukotrienes by 5-lipoxygenase (5-LO) (Murphy and Gijon 2007). By inhibiting COX-2, arachidonic acid can be shunted into the 5-LO pathway to produce abnormally high levels of leukotrienes (Hudson et al. 1993, Martel-Pelletier et al. 2004, Marcouiller et al. 2005). Conversely, ablation of 5-LO function can lead to abnormally high levels of prostaglandins (Byrum et al. 1997). Since COX-2 activity is critical for successful fracture healing, loss of COX-2 activity may alter fracture callus prostaglandin levels and lead to excess leukotriene synthesis. In turn, these excess amounts of leukotrienes could contribute to the impaired healing observed in mice lacking COX-2 or in animals treated with COX-2 inhibitors by altering the inflammatory response.

To test this hypothesis, we measured fracture healing in mice homozygous for a targeted mutation in the 5-LO gene (5-LO^KO^ mice) as loss of 5-LO activity would prevent leukotriene synthesis and potentially lead to accelerated healing. Fracture healing was assessed by radiography, histomorphometry, and torsional mechanical testing. Levels of fracture callus prostaglandin (PG) E_2_, PGF_2α_ and leukotriene (LT) B_4_ were measured to determine how loss of COX-1, COX-2, or 5-LO activity alters the synthesis of these lipid signaling molecules during fracture healing.

## Materials and methods

### Animal procedures

All animal experiments were approved by the Institutional Animal Care and Use Committee of New Jersey Medical School. Experimental mice homozygous for inactivating mutations in the 5-lipoxygenase, cyclooxygenase-1, or cyclooxygenase-2 gene (5-LO^KO^, COX-1^KO^, and COX-2^KO^, respectively) were obtained from breeding colonies. Founder mice were obtained from Jackson Laboratories (*Alox5^tm1Fun^* (5-LO^KO^); Bar Harbor, ME) or from Taconic (*Ptgs1^tm1Unc^* and *Ptgs2^tm1Unc^* (COX-1^KO^ and COX-2^KO^); Germantown, NY) (Chen et al. 1994, Langenbach et al. 1995, Morham et al. 1995). The 5-LO^KO^ mice had been backcrossed into the C57BL/6 background and so C57BL/6 mice purchased from Jackson Laboratories were used as the control for the 5-LO^KO^ mice. The COX-1^KO^ and COX-2^KO^ mice have a B6;129P2 mixed genetic background and so wild-type (B6;129P2) and COX-2 heterozygous (COX-2^Het^) mice obtained from the COX-2^KO^ breeding colony were used as controls for the COX-1^KO^ and COX-2^KO^ strains. Mouse genotypes were confirmed by PCR analysis of tail biopsy DNA samples using recombinant Pwo polymerase (Dabrowski and Kur 1998). Experiments were performed on female mice only. Mice were 10–12 weeks old at the time of fracture.

Closed femur fractures were produced in the right hind limb of mice using an established procedure (Manigrasso and O'Connor 2004). Mice were anesthetized with a mixture of ketamine and xylazine. A 0.254-mm diameter stainless steel pin was inserted retrograde into the femoral canal and wedged in place with the tip of a 0.255-mm diameter needle. The fracture was produced using a custom-made 3-point bending device. The mice were allowed to ambulate freely following fracture and were provided food and water ad libitum. They were killed with an inhaled overdose of halothane anesthesia.

### Torsional mechanical testing

The mechanical strength of the mouse femurs was determined by torsional mechanical testing to failure as described previously (Manigrasso and O'Connor 2004). Testing was accomplished using a servohydraulic testing machine (MTS Test Star, Eden Prairie, MN), a 20-Nm reaction torque cell (Interface, Scottsdale, AZ), at an actuator head displacement rate of 1 degree per second. Peak torque, rigidity, maximum shear stress, and shear modulus were calculated from the torque-displacement curves and callus dimensions as previously described (Manigrasso and O'Connor 2004). Femur cortical bone thickness was measured from cross sections of intact femurs from C57BL/6 (0.166 (0.004) mm, n = 9) and 5-LO^KO^ (0.174 (0.003), n = 9) that were approximately 14 weeks old. The fractured and contralateral femurs from each mouse were tested at 4 and 12 weeks after fracture. Values for each fractured femur were normalized to the corresponding contralateral femur as a percentage. The normalized values for each parameter were compared between the 5-LO^KO^ and C57BL/6 mice using Student's t-tests at each time point.

### Radiography, histology, and histomorphometry

All animals were examined by radiography immediately after fracture to ensure fracture quality, and after killing to assess healing and any morbid conditions. Three 5-LO^KO^ and three C57BL/6 mice were examined by radiography periodically throughout healing. Radiographs were taken using a Model 804 Packard Faxitron (Field Emission Corp., McMinnville, OR) and Kodak MINR2000 mammography film (Eastman Kodak, Rochester, NY).

For histology and histomorphometry, fractured femurs were embedded in polymethylmethacrylate using standard procedures (Baron et al. 1983). Specimens were sectioned longitudinally using a diamond wafering saw and polished with alumina grit prior to staining with van Gieson's picrofuchsin (where mineralized tissue stains red) and Stevenel's blue (where proteoglycan stains deep blue) (Maniatopoulos et al. 1986). Digital images of each specimen were captured and analyzed using Image Pro software version 5 (Media Cybernetics, Bethesda, MD). Callus, cartilage, and mineralized tissue area were measured. The cartilage and mineralized tissue area were normalized to callus area as a percentage for each specimen. Data for callus area, per cent cartilage, and per cent mineralized tissue were initially analyzed by 2-way ANOVA using genotype and time after fracture as the independent variables. The analysis found differences for callus area (p = 0.02), per cent cartilage (p < 0.001), and per cent mineralized tissue (p = 0.04). Fisher's least significant difference tests were used for post-hoc comparisons between genotypes and time points for callus area, per cent cartilage, and per cent mineralized tissue.

### Fracture callus eicosanoid levels

To determine how loss of COX-1, COX-2, and 5-LO activity affects eicosanoid synthesis during fracture healing, callus eicosanoid levels were measured 4 days after fracture during the inflammatory phase of healing. To inhibit all cyclooxygenase activity, some COX-1^KO^ and COX-2^KO^ mice were treated, respectively, with the COX-2 selective inhibitor rofecoxib (30 mg/kg VIOXX; Merck, West Point, PA), or the COX-1 selective inhibitor SC-560 (30 mg/kg; Cayman Chemicals, Ann Arbor, MI) by oral gavage using 1% methylcellulose as carrier 2 h before killing on day 4 after fracture (Smith et al. 1998, Chan et al. 1999). Each fracture callus was rapidly resected and flash frozen in liquid nitrogen. Eicosanoids were isolated by extracting the pulverized sample into M-PER buffer (Pierce Biotechnology, Inc., Rockford, IL) and then purifying the eicosanoid fraction by sequential methanol and C18 solid phase extractions as described previously (Simon and O'Connor 2007). PGE_2_, PGF_2α_, and LTB_4_ levels were measured using enzyme-linked immunoassays (Cayman Chemicals) and normalized to protein levels. Extracted protein concentration was determined using the bicinchoninic acid assay method and an aliquot of the M-PER extract (Smith et al. 1985). Values for the C57BL/6 and 5-LO^KO^ samples were compared using Student's t-tests because of genetic background differences with the cyclooxygenase-deficient strains, which includes loss of secreted phospholipase A_2_ activity in C57BL/6 mice (Kennedy et al. 1995). Values for the other strains and the rofecoxib and SC-560 treatment groups were tested for significance using ANOVA (p < 0.001) before using Fisher's least significant difference tests to identify differences between strains and the rofecoxib and SC-560 treatment groups.

## Results

### Accelerated and enhanced fracture healing in 5-LOKO mice

**Figure 1.**
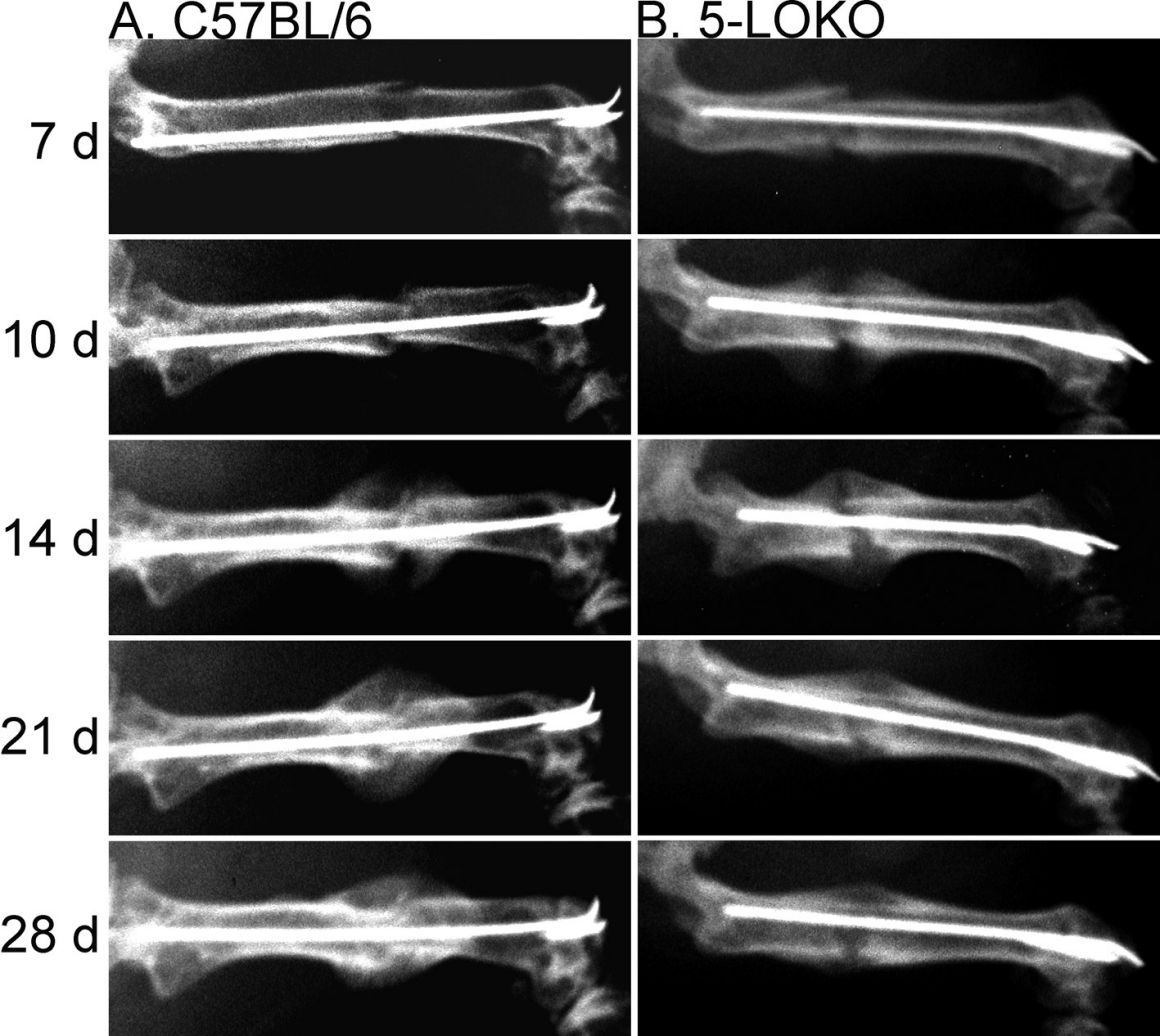
Radiographic examination of fracture healing in 5-LO^KO^ mice and control C57BL/6 mice. Shown are serial dorsal-ventral radiographs made from a C57BL/6 mouse (column A) and a 5-LO^KO^ mouse (column B) of identical age and genetic background at 7, 10, 14, 21, and 28 days after fracture, as indicated.

Fracture healing was initially assessed in the 5-LO^KO^ mice by radiography (Figure 1). We observed a mineralized callus at the periphery of the fracture site by 7 days after fracture in the 5-LO^KO^ mice as compared to 10 days in the C57BL/6 mice. After 10 days of healing, a large mineralized callus was apparent in the 5-LO^KO^ mice, which was similar in appearance to the 14-day post-fracture callus of the C57BL/6 mice. Fracture bridging was apparent by 14 days after fracture in the 5-LO^KO^ mice but not until 21 days after fracture in the C57BL/6 mice. Fracture callus size appeared reduced after 21 days of healing in the 5-LO^KO^ mice as compared to 28 days in the C57BL/6 mice. While the temporal pattern of fracture healing in the C57BL/6 mice was similar to what was previously observed in outbred (ICR) and inbred (C57BL/6, DBA/2, and C3H) strains of mice, healing appeared to be accelerated in the 5-LO^KO^ mice (Manigrasso and O'Connor 2004, Manigrasso and O'Connor 2008).

**Figure 2.**
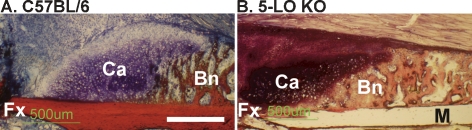
Fractures heal by endochondral ossification in 5-LO^KO^ mice. Shown are fracture callus specimens from control C57BL/6 mice (A), and 5-LO^KO^ mice (B) 7 days after fracture. Specimens were embedded in polymethylmethacrylate, sectioned, and stained with Stevenel's blue and van Gieson's picrofuchsin to identify mineralized tissue (Bn; red) and proteoglycan-rich cartilage (Ca; deep blue). The fracture site (Fx) is in the lower, left quadrant of each image. Digital images were collected at the same magnification and the white bar in panel A corresponds to 500 μm. In the 5-LO^KO^ specimen, a portion of the femoral cortical bone was lost during preparation (M).

**Figure 3.**
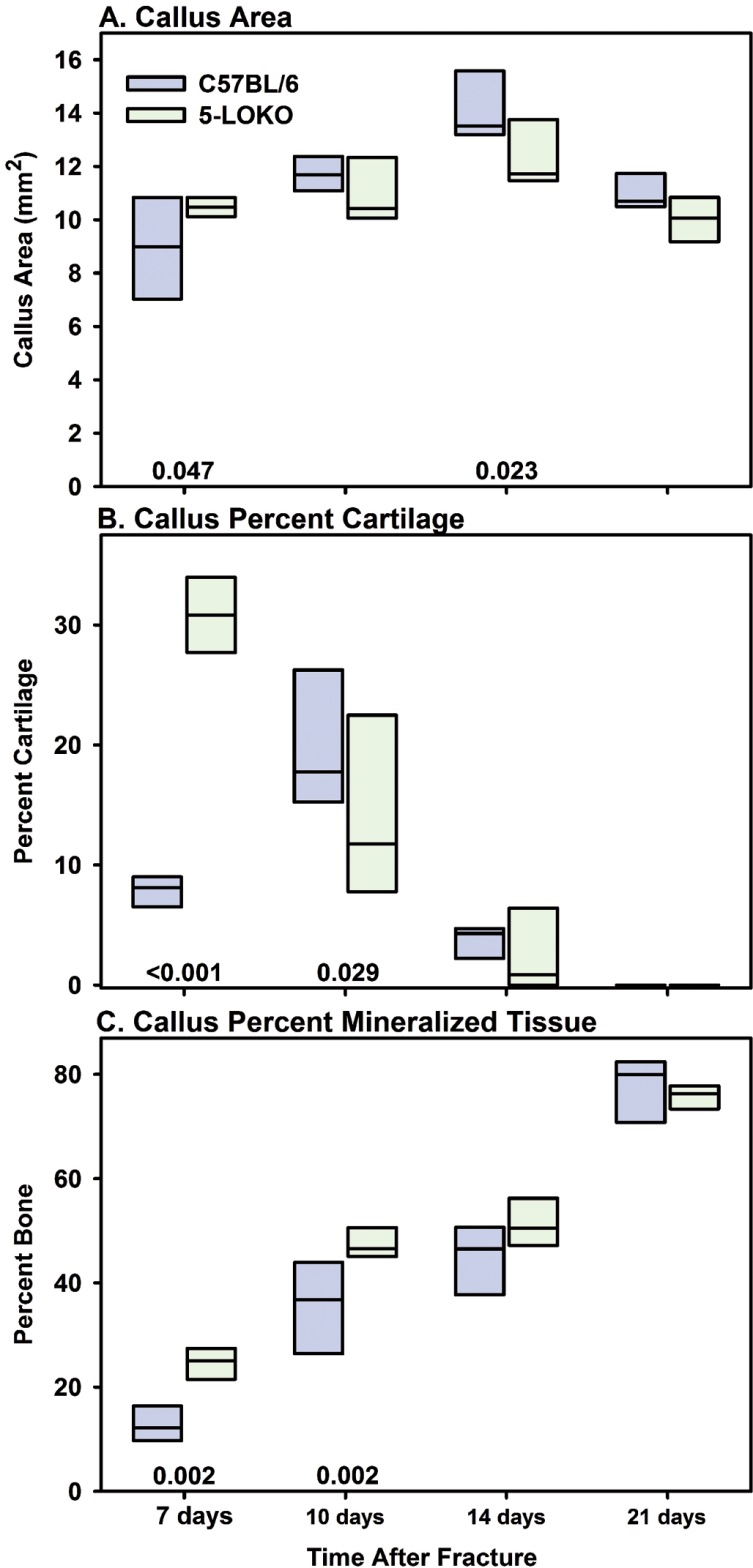
Endochondral ossification is accelerated in 5-LO^KO^ mice. Callus area (A), percent cartilage (B), and percent mineralized tissue (C) were measured for femoral fracture specimens at 7, 10, 14, and 21 days after fracture. Group size was 5 at each time point for the C57BL/6 strain (blue) and 5-LO^KO^ strain (green). Each rectangle represents the 25th and 75th percentiles and the median value is indicated with a line. Differences between the C57BL/6 and 5-LO^KO^ samples are indicated with the corresponding p-values above the x-axis.

Fractured femurs from C57BL/6 and 5-LO^KO^ mice were harvested at 7, 10, 14, and 21 days after fracture for histomorphometric analysis (n = 5 for each genotype at each time point). Callus morphology appeared relatively normal in the 5-LO^KO^ mice, with cartilage juxtaposed between the fracture site and newly formed bone at the periphery of the callus (Figure 2). These observations indicate that fractures heal via endochondral ossification in 5-LO^KO^ mice. For both mouse strains, callus area peaked at 14 days after fracture (p < 0.005 vs. 21 days) (Figure 3A). The proportion of callus cartilage in the 5-LO^KO^ mice was almost 4 times greater than that from the C57BL/6 mice at 7 days after fracture (Figure 3B). By 10 days after fracture, the proportion of callus cartilage declined in the 5-LO^KO^ mice, but peaked in the C57BL/6 mice. Further reductions in callus per cent cartilage were noted at 14 and 21 days after fracture for both mouse strains. The proportion of callus mineralized tissue was almost 2-fold higher in the 5-LO^KO^ mice at 7 days after fracture and remained higher at 10 days after fracture as compared to the C57BL/6 samples (Figure 3C). By 14 and 21 days after fracture, no statistically significant difference in the percentage of callus mineralized tissue was found between the mouse strains. The data indicate that bone regeneration occurs through an accelerated endochondral ossification process in the 5-LO^KO^ mice.

**Figure 4.**
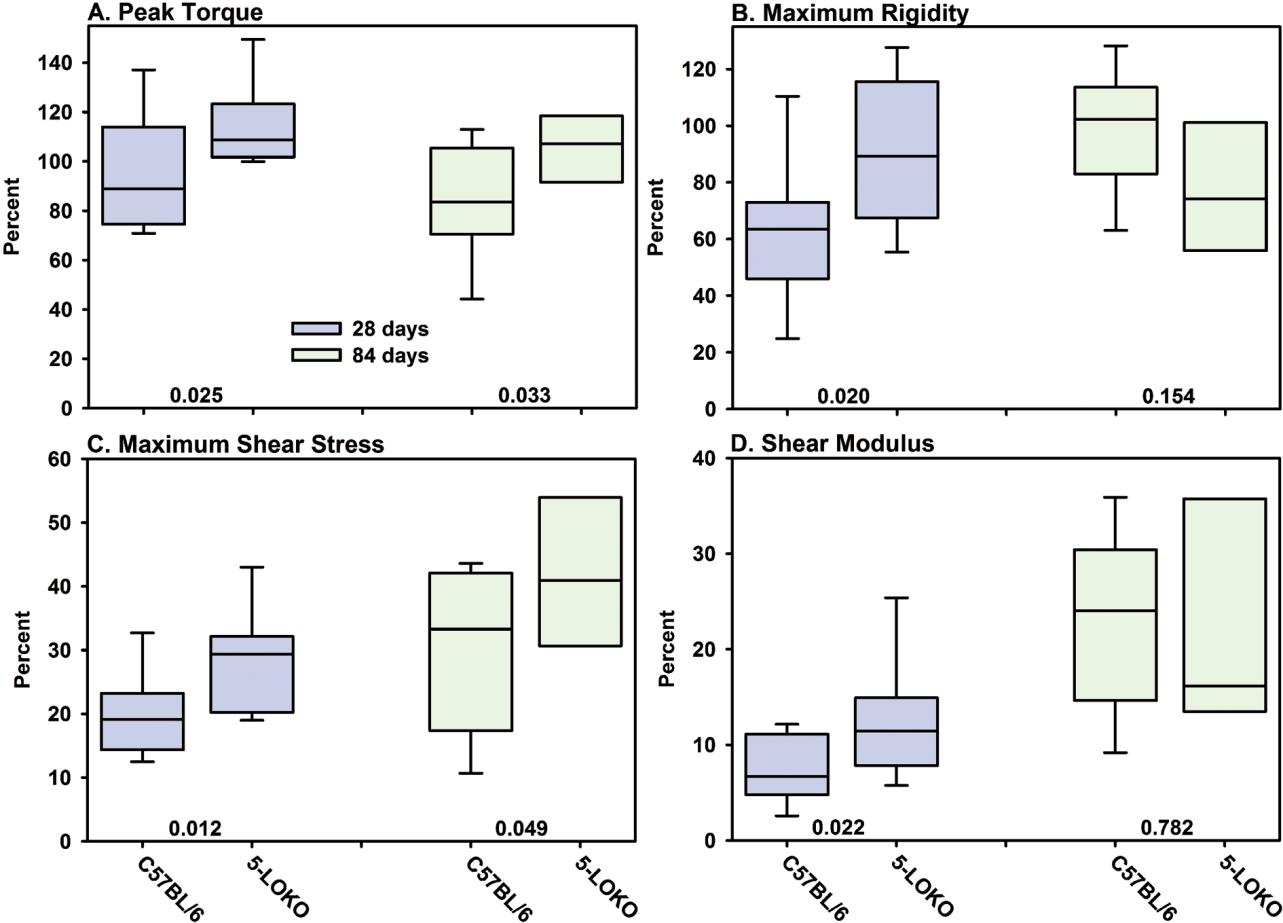
Fracture calluses from 5-LO^KO^ mice have enhanced mechanical properties. Fractured and contralateral femurs after 28 days (blue) and 84 days (green) of healing from control C57BL/6 mice (n = 11 at 28 and 84 days) and 5-LO^KO^ mice (n = 11 and 8 at 28 and 84 days, respectively) were mechanically tested to failure in torsion. Peak torque (panel A), maximum rigidity (panel B), maximum shear stress (panel C), and shear modulus (panel D) were calculated from the torque to angular displacement curves and callus dimensions. Fractured femur values were normalized to values from the contralateral femur of that mouse as a percentage. The limits of each rectangle represent the 25th and 75th percentile of the normalized values. The median value is indicated in each rectangle. The 5th and 95th percentile values are shown below and above each rectangle for those with group sizes of 11. Normalized values were compared between genotypes at each time point and p-values are indicated in each graph above the x-axis.

Fracture callus mechanical properties were measured in the 5-LO^KO^ and C57BL/6 mice after 4 and 12 weeks of healing (Figure 4). Contralateral femurs from each mouse were also mechanically tested to failure in torsion (Table). Despite having the same genetic background, femur morphology was different between the C57BL/6 and 5-LO^KO^ strains (Table). The better structural properties of the unfractured, contralateral C57BL/6 femurs probably relates to the larger diameter of these bones as compared to their 5-LO^KO^ counterparts. Because of the baseline differences in femoral mechanical properties, fracture callus mechanical properties were normalized to the contralateral femur values as a percentage. Normalized peak torque and maximum rigidity of the 5-LO^KO^ fracture calluses were approximately 20% and 40% higher than the C57BL/6 values after 4 weeks of healing. Similarly, normalized maximum shear stress and shear modulus were approximately 40% and 70% higher in the 5-LO^KO^ mice after 4 weeks of healing. By 12 weeks, normalized peak torque and maximum shear stress remained higher in the 5-LO^KO^ mouse fractures than in the C57BL/6 mice. The mechanical testing data demonstrate that the 5-LO^KO^ fracture callus regains structural and material properties sooner than the fracture callus in control C57BL/6 mice.

**Table:** Contralateral mechanical values for unfractured femurs

A	B	C	D	E	F	G	H	I	J
C57BL/6	14–16	11	14.67 (0.26)	1.73 (0.07)	1.20 (0.05)	31.9 (5.0)	819 (200)	144 (30)	4.2 (1.0)
5-LO^KO^	14–16	11	14.30 (0.21)	1.64 (0.08)	1.16 (0.05)	24.8 (4.9)	688 (168)	126 (28)	4.2 (1.0)
p-value	—	—	0.002	0.011	0.077	0.003	0.111	0.176	0.997
C57BL/6	22–24	11	15.26 (0.21)	1.77 (0.10)	1.23 (0.04)	37.2 (8.4)	784 (208)	156 (37)	3.8 (1.2)
5-LO^KO^	22–24	8	15.11 (0.39)	1.53 (0.07)	1.17 (0.03)	23.8 (5.7)	645 (157)	130 (29)	4.5 (0.8)
p-value	—	—	0.293	< 0.001	0.002	0.001	0.131	0.117	0.130

A Genotype B Age (weeks) C Sample size D Length (mm) E Max. diameter (mm) F Min. diameter (mm) G Peak torque (Nmm) H Max. rigidity (Nmm^2^/rad) I Max. shear stress (MPa) J Shear modulus (Ga)

### Fracture callus eicosanoid levels in COX-1^KO^, COX-2^KO^, and 5-LO^KO^ mice

**Figure 5.**
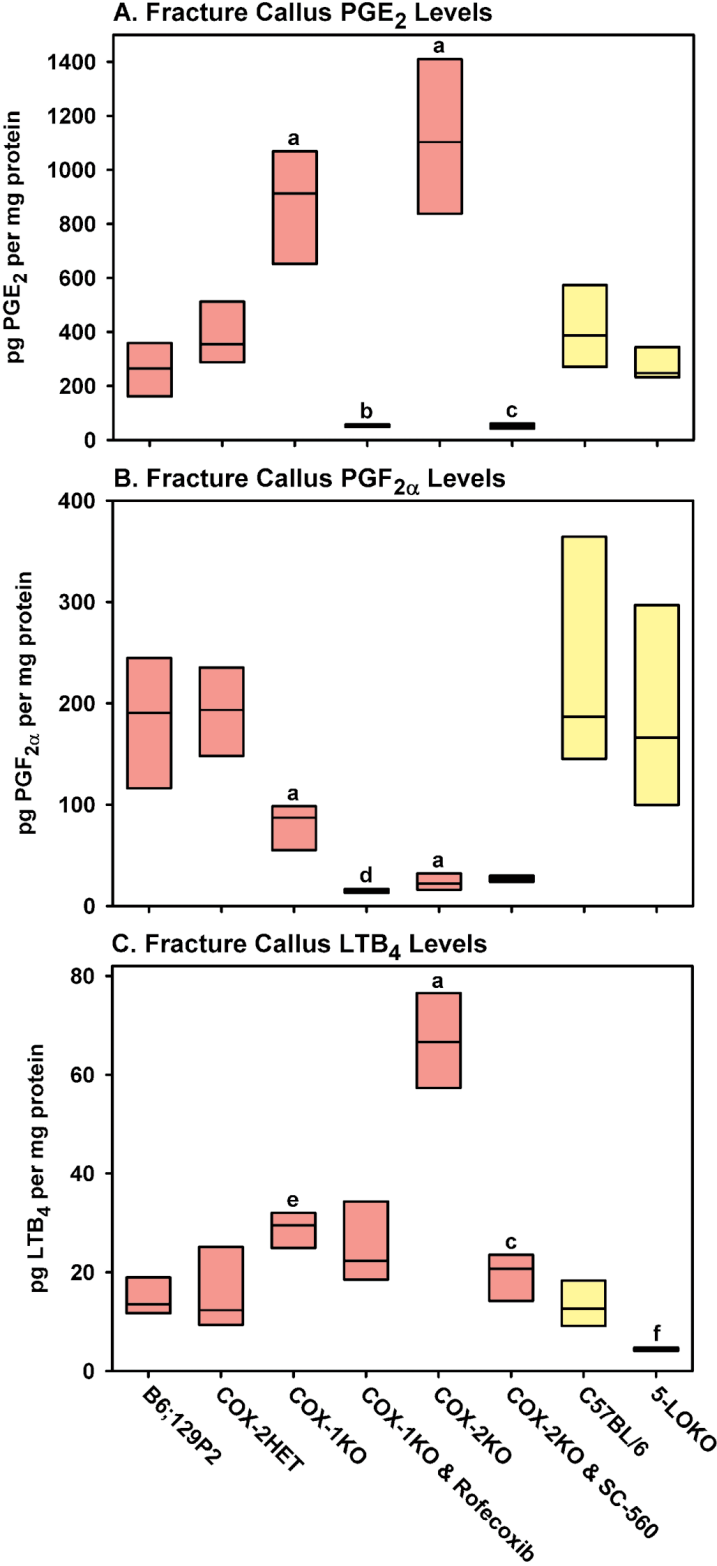
Loss of COX-1, COX-2, or 5-LO function alters callus eicosanoid levels. Rectangles represent the 25th and 75th percentiles with median values shown for fracture callus PGE_2_ levels (A), PGF_2α_ levels (B), and LTB_4_ levels (C) measured 4 days after fracture in control B6;129P2 mice (n = 6), COX-2^HET^ mice (n = 6), COX-1^KO^ mice (n = 4), COX-1^KO^ mice treated with 30 mg/kg of rofecoxib (n = 3), COX-2^KO^ mice (n = 6), COX-2^KO^ mice treated with 30 mg/kg of SC-560 (n = 5), control C57BL/6 mice (n = 6), and 5-LO^KO^ mice (n = 6). Significant differences are indicated as a, p < 0.001 vs. COX-2^HET^; b, p < 0.001 vs. COX-1^KO^; c, p < 0.001 vs. COX-2^KO^; d, p = 0.04 vs. COX-1^KO^; e, p = 0.02 vs COX-2^HET^; and f, p = 0.002 vs. C57BL/6. Red bars indicate values from mice with the mixed B6;129P2 background and yellow bars indicate values from mice with the C57BL/6 background.

The effects of lost COX-1, COX-2, and 5-LO activity on PGE_2_, PGF_2α_, and LTB_4_ synthesis was measured at day 4 after fracture (Figure 5). Surprisingly, callus PGE_2_ levels were more than 3-fold higher in the COX-1^KO^ and COX-2^KO^ callus samples than in the COX-2^HET^ samples (Figure 5A). Treatment of the COX-1^KO^ mice with rofecoxib to inhibit COX-2 dramatically reduced callus PGE_2_ levels and, similarly, treatment of the COX-2^KO^ mice with SC-560 to inhibit COX-1 dramatically reduced callus PGE_2_ levels. No statistically significant effect on callus PGE_2_ levels was seen in the 5-LO^KO^ mice (p = 0.1 vs. C57BL/6).

In contrast to PGE_2_, callus PGF_2α_ levels were significantly lower in the COX-2^KO^ mice as compared to the other mouse strains, and treatment with SC-560 to inhibit COX-1 caused no further reduction in PGF_2α_ levels (p = 0.9). Callus PGF_2α_levels were also reduced in the COX-1^KO^ mice, but they were also higher than in the COX-2^KO^ mice (p = 0.04). Treatment of COX-1^KO^ mice with rofecoxib to inhibit COX-2 further reduced callus PGF_2α_ levels (p = 0.04) to values similar to those found in the COX-2^KO^ mice (p = 0.7). No significant effect on callus PGF_2α_ level was detected in the 5-LO^KO^ mice (p = 0.4 vs. C57BL/6).

As expected, callus LTB_4_ levels were low in the 5-LO^KO^ mice. Conversely, they were almost 2-fold higher in the COX-1^KO^ callus samples (p = 0.02) than in the B6;129P2 samples, while COX-2^KO^ callus LTB4 levels were over 4-fold higher than in the B6;129P2 samples (p < 0.001) and over 2-fold higher than in the COX-1^KO^ samples (p < 0.001). Treatment of COX-1^KO^ mice with rofecoxib had no significant effect on callus LTB_4_ levels, but treatment of the COX-2^KO^ mice with SC-560 caused a 3.5-fold decrease in callus LTB_4_ levels.

## Discussion

The normal functions of 5-LO in bone metabolism are not well described. Genetic loss of 5-LO activity alters bone morphology (Table) and increases cortical bone thickness (Bonewald et al. 1997). Leukotrienes produced via 5-LO can stimulate osteoclast activity, suggesting that the increased cortical thickness found in 5-LO^KO^ mice may be associated with reduced osteoclast activity (Gallwitz et al. 1993, Garcia et al. 1996, Flynn et al. 1999).

Our findings indicate that endochondral ossification during fracture healing is accelerated in 5-LO^KO^ mice, leading to reduced bridging time and enhanced biomechanical properties of the callus (Figures 1-4). The fracture calluses of the 5-LO^KO^ mice obtained greater maximum shear stress and shear modulus sooner than the C57BL/6 controls. Whether the enhanced material properties are a direct consequence of lost 5-LO activity or an indirect consequence of earlier bridging remains to be determined. These experimental results support the hypothesis that 5-LO metabolites such as LTB_4_ can impede fracture healing. Indeed, in vitro studies have shown that LTB_4_ treatment can impede osteoblast activity (Ren and Dziak 1991, Traianedes et al. 1998).

Measurement of eicosanoid levels in fracture callus supports the initial hypothesis that loss of COX-2 activity can lead to shunting of arachidonic acid into the 5-LO pathway, since 4-fold more LTB_4_ was found in the COX-2^KO^ fracture callus (67 pg/mg of protein) than in the B6;129P2 controls (15 pg/mg of protein) (Figure 5). While the 2-fold increase in COX-1^KO^ callus LTB_4_ (29 pg/mg of protein) also supports an arachidonic acid shunting mechanism, the magnitude of callus LTB_4_ levels in the COX-1^KO^ mice may not be sufficient to impair fracture healing. Unexpectedly, callus PGE_2_ levels were dramatically higher in the COX-1^KO^ callus (3-fold) and COX-2^KO^ callus (4-fold) than in the B6;129P2 controls. Thus, impaired fracture healing in COX-2^KO^ mice does not appear to be caused by lack of PGE_2_. This suggests that therapies designed to activate the EP_2_ or EP_4_ receptors for PGE_2_ may be enhancing healing by a non-specific mechanism (Li et al. 2003, 2005, Paralkar et al. 2003, Tanaka et al. 2004).

Perhaps more intriguing was the observation that PGF_2α_ synthesis appears to be dependent upon COX-2 activity. This suggests that COX-2-dependent PGF_2α_ synthesis may be critical for endochondral ossification during fracture healing. Indeed, glycosaminoglycan synthesis and the expression of type II collagen and aggrecan were stimulated in human articular chondrocyte pellet cultures treated with PGF_2α_, to a lesser extent by PGD_2_, but not by PGE_2_ (Jakob et al. 2004). PGF_2α_ treatment also stimulated replication and sulfate incorporation by rat RCJ3.1C5.18 chondrocytes at levels 10–100 fold lower than PGE_2_ (Lowe et al. 1996). Analysis of tissue prostaglandin levels during demineralized bone matrix-induced heterotopic ossification in rats showed that chondrocyte differentiation and progression into hypertrophy correlated with peak PGF_2α_ levels (Wientroub et al. 1983). Thus, lack of PGF_2α_ correlates well with impaired fracture healing in COX-2^KO^ mice. Unlike COX-2^KO^ mice, PGF_2α_ levels in COX-1^KO^ mice appear to be sufficient since fracture healing proceeds normally in COX-1^KO^ mice (Simon et al. 2002). Why PGF_2α_ levels were lower in the COX-1^KO^ callus as compared to the B6;129P2 controls but higher than in COX-2^KO^ callus is not known. The differences could relate to preferential coupling of PGF_2α_ synthase to heterodimers of COX-1 and COX-2 as compared to COX-2 homodimers (which would be the only available form of cyclooxygenase in COX-1^KO^ mice), and failure of PGF_2α_ synthase to couple with COX-1 homodimers (which would be the only available cyclooxygenase form in COX-2^KO^ mice) (Yu et al. 2006, Yuan et al. 2006). Genetic ablation of COX-1 or COX-2 may also alter the temporal pattern of prostaglandin synthesis. We only measured prostaglandin levels at a single time point after fracture; additional experiments are needed to extend these results and confirm when and to what level the synthesis of each prostaglandin peaks during fracture healing. It is likely that the bioactive lipids synthesized via COX-2 and 5-LO have a multiplicity of effects during tissue regeneration or repair.

The cell types that synthesize and respond to prostaglandins or leukotrienes during bone healing are not known. Prostaglandins can affect osteoblasts, chondrocytes, and osteoclasts (Raisz 1999, Zhang et al. 2004, Clark et al. 2005). It has also been suggested that prostaglandins can affect fracture callus mesenchymal cells (Zhang et al. 2002). Much less is known about effects of leukotrienes on these cell types (Ren and Dziak 1991, Traianedes et al. 1998). Prostaglandins also have proangiogenic effects; thus, reduced angiogenesis may underlie—at least in part—the impaired fracture healing response caused by loss of COX-2 function (Form and Auerbach 1983, Seno et al. 2002). The expression of 5-LO and synthesis of leukotrienes occurs primarily in cells of the myeloid lineage (Steinhilber 1994). However, 5-LO expression in primary cultures of human osteoblasts has been detected (Maxis et al. 2006). It is likely that the bioactive lipids synthesized via COX-2 and 5-LO have multiple effects during tissue regeneration or repair.

Our study shows that 5-LO negatively regulates fracture healing. In contrast, previous studies have shown that COX-2 is a positive regulator of fracture healing (Simon et al. 2002, Simon and O'Connor 2007). Thus, two enzymes that use the same lipid substrate to produce different bioactive lipids have diametric effects on bone regeneration. Consistent with these results, inhibition of 5-LO with an orally delivered drug has been shown to accelerate and enhance fracture healing in another animal model (Cottrell and O'Connor 2009). Pharmacological or genetic manipulation of the arachidonic acid metabolic and signaling pathways to alter COX-2 and 5-LO activity may be a means to accelerate and enhance tissue repair or regeneration.

## References

[CIT0001] Baron R, Vigney A, Neff L, Silvergate A, Santa Maria A, Recker RR (1983). Processing of undecalcified bone specimens for bone histomorphometry. Bone histomorphometry: Techniques and hnterpretation.

[CIT0002] Bonewald LF, Flynn M, Qiao M, Dallas MR, Mundy GR, Boyce BF (1997). Mice lacking 5-lipoxygenase have increased cortical bone thickness. Adv Exp Med Biol.

[CIT0003] Burd TA, Hughes MS, Anglen JO (2003). Heterotopic ossification prophylaxis with indomethacin increases the risk of long-bone nonunion. J Bone Joint Surg (Br).

[CIT0004] Byrum RS, Goulet JL, Griffiths RJ, Koller BH (1997). Role of the 5-lipoxygenase-activating protein (FLAP) in murine acute inflammatory responses. J Exp Med.

[CIT0005] Capper EA, Marshall LA (2001). Mammalian phospholipases A(2): mediators of inflammation. proliferation and apoptosis. Prog Lipid Res.

[CIT0006] Chan C-C, Boyce S, Brideau C, Charleson S, Cromlish W, Ethier D (1999). Rofecoxib [Vioxx, MK-0966; 4-(4'-methylsulfonylphenyl)-3-phenyl-2-(5H)-furanone]: a potent and orally active cyclooxygenase-2 inhibitor. Pharmacological and biochemical profiles. J Pharmacol Exp Ther.

[CIT0007] Chen XS, Sheller JR, Johnson EN, Funk CD (1994). Role of leukotrienes revealed by targeted disruption of the 5-lipoxygenase gene. Nature.

[CIT0008] Clark CA, Schwarz EM, Zhang X, Ziran NM, Drissi H, O'Keefe RJ (2005). Differential regulation of EP receptor isoforms during chondrogenesis and chondrocyte maturation. Biochem Biophys Res Commun.

[CIT0009] Cottrell JA, O'Connor JP (2009). Pharmacological inhibition of 5-lipoxygenase accelerates and enhances fracture-healing. J Bone Joint Surg (Am).

[CIT0010] Cottrell JA, Meyenhofer M, Medicherla S, Higgins L, O'Connor JP (2009). Analgesic effects of p38 kinase inhibitor treatment on bone fracture healing. Pain.

[CIT0011] Dabrowski S, Kur J (1998). Cloning and expression in Escherichia coli of the recombinant his-tagged DNA polymerases from Pyrococcus furiosus and Pyrococcus woesei. Protein Expr Purif.

[CIT0012] Flynn MA, Qiao M, Garcia C, Dallas M, Bonewald LF (1999). Avian osteoclast cells are stimulated to resorb calcified matrices by and possess receptors for leukotriene B4. Calcif Tissue Int.

[CIT0013] Form DM, Auerbach R (1983). PGE2 and angiogenesis. Proc Soc Exp Biol Med.

[CIT0014] Gallwitz WE, Mundy GR, Lee CH, Qiao M, Roodman GD, Raftery M (1993). 5-Lipoxygenase metabolites of arachidonic acid stimulate isolated osteoclasts to resorb calcified matrices. J Biol Chem.

[CIT0015] Garcia C, Boyce BF, Gilles J, Dallas M, Qiao M, Mundy GR (1996). Leukotriene B4 stimulates osteoclastic bone resorption both in vitro and in vivo. J Bone Miner Res.

[CIT0016] Hudson N, Balsitis M, Everitt S, Hawkey CJ (1993). Enhanced gastric mucosal leukotriene B4 synthesis in patients taking non-steroidal anti-inflammatory drugs. Gut.

[CIT0017] Jakob M, Demarteau O, Suetterlin R, Heberer M, Martin I (2004). Chondrogenesis of expanded adult human articular chondrocytes is enhanced by specific prostaglandins. Rheumatology (Oxford).

[CIT0018] Kennedy BP, Payette P, Mudgett J, Vadas P, Pruzanski W, Kwan M (1995). A natural disruption of the secretory group II phospholipase A2 gene in inbred mouse strains. J Biol Chem.

[CIT0019] Langenbach R, Morham SG, Tiano HF, Loftin CD, Ghanayem BI, Chulada PC (1995). Prostaglandin synthase 1 gene disruption in mice reduces arachidonic acid-induced inflammation and indomethacin-induced gastric ulceration. Cell.

[CIT0020] Li M, Ke HZ, Qi H, Healy DR, Li Y, Crawford DT (2003). A novel, non-prostanoid EP2 receptor-selective prostaglandin E2 agonist stimulates local bone formation and enhances fracture healing. J Bone Miner Res.

[CIT0021] Li M, Healy DR, Li Y, Simmons HA, Crawford DT, Ke HZ (2005). Osteopenia and impaired fracture healing in aged EP4 receptor knockout mice. Bone.

[CIT0022] Lowe GN, Fu YH, McDougall S, Polendo R, Williams A, Benya PD (1996). Effects of prostaglandins on deoxyribonucleic acid and aggrecan synthesis in the RCJ 3.1C5.18 chondrocyte cell line: role of second messengers. Endocrinology.

[CIT0023] Maniatopoulos C, Rodriguez A, Deporter DA, Melcher AH (1986). An improved method for preparing histological sections of metallic implants. Int J Oral Maxillofac Implants.

[CIT0024] Manigrasso MB, O'Connor JP (2004). Characterization of a closed femur fracture model in mice. J Orthop Trauma.

[CIT0025] Manigrasso MB, O'Connor JP (2008). Comparison of fracture healing among different inbred mouse strains. Calcif Tissue Int.

[CIT0026] Marcouiller P, Pelletier JP, Guevremont M, Martel-Pelletier J, Ranger P, Laufer S (2005). Leukotriene and prostaglandin synthesis pathways in osteoarthritic synovial membranes: regulating factors for interleukin 1beta synthesis. J Rheumatol.

[CIT0027] Martel-Pelletier J, Mineau F, Fahmi H, Laufer S, Reboul P, Boileau C (2004). Regulation of the expression of 5-lipoxygenase-activating protein/5-lipoxygenase and the synthesis of leukotriene B(4) in osteoarthritic chondrocytes: role of transforming growth factor beta and eicosanoids. Arthritis Rheum.

[CIT0028] Maxis K, Delalandre A, Martel-Pelletier J, Pelletier JP, Duval N, Lajeunesse D (2006). The shunt from the cyclooxygenase to lipoxygenase pathway in human osteoarthritic subchondral osteoblasts is linked with a variable expression of the 5-lipoxygenase-activating protein. Arthritis Res Ther.

[CIT0029] Morham SG, Langenbach R, Loftin CD, Tiano HF, Vouloumanos N, Jennette JC (1995). Prostaglandin synthase 2 gene disruption causes severe renal pathology in the mouse. Cell.

[CIT0030] Mountziaris PM, Mikos AG (2008). Modulation of the inflammatory response for enhanced bone tissue regeneration. Tissue Eng Part B Rev.

[CIT0031] Murphy RC, Gijon MA (2007). Biosynthesis and metabolism of leukotrienes. Biochem J.

[CIT0032] Paralkar VM, Borovecki F, Ke HZ, Cameron KO, Lefker B, Grasser WA (2003). An EP2 receptor-selective prostaglandin E2 agonist induces bone healing. Proc Natl Acad Sci U S A.

[CIT0033] Raisz LG (1999). Prostaglandins and bone: physiology and pathophysiology. Osteoarthritis Cartilage.

[CIT0034] Ren W, Dziak R (1991). Effects of leukotrienes on osteoblastic cell proliferation. Calcif Tissue Int.

[CIT0035] Seno H, Oshima M, Ishikawa TO, Oshima H, Takaku K, Chiba T (2002). Cyclooxygenase 2- and prostaglandin E(2) receptor EP(2)-dependent angiogenesis in Apc(Delta716) mouse intestinal polyps. Cancer Res.

[CIT0036] Simmons DL, Botting RM, Hla T (2004). Cyclooxygenase isozymes: the biology of prostaglandin synthesis and inhibition. Pharmacol Rev.

[CIT0037] Simon AM, Manigrasso MB, O'Connor JP (2002). Cyclo-oxygenase 2 function is essential for bone fracture healing. J Bone Miner Res.

[CIT0038] Simon AM, O'Connor JP (2007). Dose and time-dependent effects of cyclooxygenase-2 inhibition on fracture-healing. J Bone Joint Surg (Am).

[CIT0039] Smith PK, Krohn RI, Hermanson GT, Mallia AK, Gartner FH, Provenzano MD (1985). Measurement of protein using bicinchoninic acid. Anal Biochem.

[CIT0040] Smith CJ, Zhang Y, Koboldt CM, Muhammad J, Zweifel BS, Shaffer A (1998). Pharmacological analysis of cyclooxygenase-1 in inflammation. Proc Natl Acad Sci U S A.

[CIT0041] Steinhilber D (1994). 5-Lipoxygenase: enzyme expression and regulation of activity. Pharm Acta Helv.

[CIT0042] Tanaka M, Sakai A, Uchida S, Tanaka S, Nagashima M, Katayama T (2004). Prostaglandin E2 receptor (EP4) selective agonist (ONO-4819.CD) accelerates bone repair of femoral cortex after drill-hole injury associated with local upregulation of bone turnover in mature rats. Bone.

[CIT0043] Traianedes K, Dallas MR, Garrett IR, Mundy GR, Bonewald LF (1998). 5-Lipoxygenase metabolites inhibit bone formation in vitro. Endocrinology.

[CIT0044] Wientroub S, Wahl LM, Feuerstein N, Winter CC, Reddi AH (1983). Changes in tissue concentration of prostaglandins during endochondral bone differentiation. Biochem Biophys Res Commun.

[CIT0045] Yu Y, Fan J, Chen XS, Wang D, Klein-Szanto AJ, Campbell RL (2006). Genetic model of selective COX2 inhibition reveals novel heterodimer signaling. Nat Med.

[CIT0046] Yuan C, Rieke CJ, Rimon G, Wingerd BA, Smith WL (2006). Partnering between monomers of cyclooxygenase-2 homodimers. Proc Natl Acad Sci U S A.

[CIT0047] Zhang X, Schwarz EM, Young DA, Puzas JE, Rosier RN, O'Keefe RJ (2002). Cyclooxygenase-2 regulates mesenchymal cell differentiation into the osteoblast lineage and is critically involved in bone repair. J Clin Invest.

[CIT0048] Zhang X, Ziran N, Goater JJ, Schwarz EM, Puzas JE, Rosier RN (2004). Primary murine limb bud mesenchymal cells in long-term culture complete chondrocyte differentiation: TGF-beta delays hypertrophy and PGE2 inhibits terminal differentiation. Bone.

